# Dietary Threonine Promoted the Growth and Ovarian Development of the Red Swamp Crayfish (*Procambarus clarkii*)

**DOI:** 10.1155/2024/5526963

**Published:** 2024-05-20

**Authors:** Haihang Yao, Manxia Cao, Jianmin Zhang, Shouqi Xie, Kai Luo, Wenfu Xiao, Lixue Dong, Weihua Gao, Juan Tian

**Affiliations:** ^1^Yangtze River Fisheries Research Institute, Chinese Academy of Fishery Sciences, Wuhan 430223, China; ^2^Hubei Key Laboratory of Waterlogging Disaster and Agricultural Use of Wetland, Yangtze University, Jingzhou 434024, China; ^3^State Key Laboratory of Freshwater Ecology and Biotechnology, Institute of Hydrobiology, Chinese Academy of Sciences, Wuhan 430072, China

## Abstract

To explore the effects of dietary threonine on growth and ovarian development of red swamp crayfish (*Procambarus clarkii*), crayfish (5.48 ± 0.19 g) were fed six isoproteic and isoenergetic diets with varying levels of threonine (7.16 g/kg (control), 9.19, 12.74, 16.44, 20.83, and 23.78 g/kg) for 8 weeks. The results showed that weight gain rate, feed conversion ratio, protein efficiency rate, protein deposition rate, and essential amino acid deposition rates obtained the optimal values when the dietary threonine level was 12.74 or 16.44 g/kg. Compared to the control group, the 12.74 g/kg group exhibited enhanced nonspecific immunity and antioxidant properties. The 16.44 g/kg group demonstrated a significant increase in the frequency of B cells and R cells in the hepatopancreas, the length and width of intestinal villi, and the activities of protease and lipase. It also showed elevated ecdysterone hormone, gonadal index (GI), cAMP content, and the relative abundance of beneficial intestinal microflora. Compared to the control group, the mRNA expression of *mTOR*, *S6K1*, *4EBP1*, *EcR*, *RXR*, *chitinase*, *PKA*, *Vg*, *cdc2*, and *cyclin B* was significantly upregulated, and the mRNA expression of *MIH* was significantly downregulated in the 16.44 g/kg group. Overall, optimal dietary threonine could improve intestinal health, regulate immune function, and enhance protein utilization, molting, and growth performance of red swamp crayfish. Additionally, it improved the synthesis of yolk substance and facilitated the development of ovarian cells of female crayfish. The optimal threonine level was 14.87–16.94 g/kg (dry matter), corresponding to 42.51–48.42 g/kg of dietary protein in red swamp crayfish.

## 1. Introduction

Essential amino acids (EAAs) are closely related to the growth, reproduction, and immunity of crustaceans [1–3]. Molting is a crucial process for the growth and reproduction of crustaceans [4]. Previous studies have shown that EAAs activate ecdysone (EH) secretion and vitellogenin (*Vg*) synthesis through the rapamycin target protein (*mTOR*) signaling pathway and the crustacean hyperglycemic hormone (CHH), promoting crustacean molting and yolk deposition [5–7]. Meanwhile, EAAs and their metabolites can regulate the nonspecific immune function of crustaceans by activating the prophenoloxidase (proPO) system [8]. Therefore, it is crucial to determine the dietary EAAs requirements for crustaceans.

Threonine (Thr) is one of the EAAs with an alcohol-group structure [3, 9] and is the third limited amino acid after lysine and methionine in the low fish meal formula diet [10]. It is essential for protein synthesis, energy metabolism, nutritional absorption, and immune function [11–13]. Currently, research on threonine in aquatic animals mainly focuses on dietary requirements, physiological functions, and metabolic regulation [14]. Dietary threonine requirements have been reported in white shrimp (*Litopenaeus vannamei*) [3, 15], Chinese mitten crab (*Eriocheir sinensis*) [16], and black tiger shrimp (*Penaeus monodon*) [17], ranging from 11.80 to 15.90 g/kg of dry matter, corresponding to 28.10–39.80 g/kg of dietary protein. Threonine (15.10 g/kg) significantly improved the growth, feed efficiency, and protein efficiency in white shrimp [3]. Dietary threonine (15.90 g/kg) improved the nonspecific immune function of juvenile Chinese mitten crab [16]. Additionally, studies in mammals have shown that threonine regulates the progesterone level and improves reproductive performance by regulating phosphorylated-*mTOR* of pregnant sows [18, 19]. Threonine deficiency occurs in the eggs of green mud crab (*Scylla paramamosain*) after continuous oviposition, suggesting that threonine may be related to the ovarian nutrient deposition of crustaceans [20]. At present, there are few studies on the effects of threonine on the ovarian development of crustaceans.

Red swamp crayfish (*Procambarus clarkii*) is favored by consumers because of its fresh meat, delicious flavor, and rich nutrients [21]. In 2022, the total output of red swamp crayfish reached 2.89 million tons, with a comprehensive output value of 458 billion yuan, and it has become an economically cultured species with high economic performance and high quality in China [22]. However, there was little research on the amino acid requirement of red swamp crayfish, particularly regarding ovarian development. This has resulted in a low feed utilization rate and poor growth performance, ultimately affecting parent selection and seedling quality. Therefore, the present study was conducted to investigate the dietary threonine requirements and ovarian development of red swamp crayfish through exploring its effects on growth performance, digestive organ function, nonspecific immunity, and ovarian development. This will be providing a technical reference for preparing high-efficiency red swamp crayfish diets and a theoretical basis for explaining the effects of threonine on the growth and ovarian development of crustaceans.

## 2. Materials and Methods

### 2.1. Ethics Statement

Red swamp crayfish is widely cultivated in China and is not listed as an endangered or protected species. All animal care and use procedures were approved by the Institutional Animal Care and Use Committee of Yangtze River Fisheries Research Institute (according to YFI 2018-40 of July 20, 2018).

### 2.2. Experimental Diets

Six isoproteic and isoenergetic diets were prepared using fish meal, wheat gluten, peanut meal, and crystal amino acids as protein sources, fish oil and soybean oil as fat sources, and flour as a carbohydrate source. Crystalline L-threonine was obtained from Shanghai Macklin Biochemical Co., Ltd. (Shanghai, purity ≥ 99%). The added levels of threonine were 0 g/kg (control), 2.50, 5.00, 7.50, 10.00, and 12.50 g/kg, respectively. Crystalline L-alanine was used to balance the dietary protein level. The amino acid concentrations of dried samples of diets (0.2 g) were determined using an automatic threonine analyzer (HITACHI L-8900, Tokyo, Japan). The actual contents of dietary threonine in six diets were 7.16, 9.19, 12.74, 16.44, 20.83, and 23.78 g/kg, respectively. The diet formula, proximate nutrient compositions, and amino acid compositions are shown in Tables 1 and 2. Our previous study has described the diet preparation method [23]. The dissolution rate of the diets was determined by dissolving dry diet in water for 2 hr and then taken out, redried, and weighted. The average result was 10.77%.

### 2.3. Experimental Animals and Feeding Management

The experimental red swamp crayfish were purchased from a commercial hatchery (Qianjiang, Hubei Province, China). The feeding experiment was conducted at the experimental base of the Yangtze River Fisheries Research Institute, Chinese Academy of Fisheries Sciences (Wuhan, China). The culture system is equipped with shielding tubes and climbing nets to facilitate molting and prevent individual fighting. All crayfish (stocking density was 60 crayfish/m^3^ water) were fed the control group diet for 1 week to adapt to the experimental diets and conditions. Before the feeding trial, crayfish were fasted for 24 hr and weighed after anesthesia with 60 mg/L eugenol. Ten crayfish were randomly selected as initial samples and stored at −40°C to evaluate the initial proximate body compositions. Crayfish (5.48 ± 0.19 g) were distributed into 18 containers (depth: 0.35 m, water volume: 350 L), with 18 crayfish per container and three containers per treatment. The six experimental diets were fed by hand twice daily at 8:00 and 18:00. Daily feeding rate was 3%–5% of the total weight of crayfish per container and was adjusted according to the feeding behavior of crayfish in the previous day. The dead crayfish and uneaten diet were weighed and recorded during the experiment. Each container was refilled daily with 300 L of fresh water at a microflow rate of 30 liters per hour. The water temperature was in the range of 23°C–28°C, the dissolved oxygen was > 5 mg/L, the pH was in the range of 8.2–8.4, and the ammonia nitrogen was < 0.05 mg/L. The feeding experiment lasted 56 days.

### 2.4. Sample Collection

At the end of the feeding experiment, the crayfish were fasted for 24 hr and anesthetized with 60 mg/L eugenol. The crayfish were counted and weighed, and the survival rate, weight gain rate, specific growth rate, and feed conversion ratio were calculated. Eight crayfish were randomly selected from each container to measure their body weight first. The hemolymph was extracted from the pericardial cavity using a sterile 1 mL syringe and placed in a 1.5 mL centrifuge tube at 4°C for 4 hr. The hemolymph was homogenized using a syringe needle to prevent clotting and then centrifuged (14,400x *g*, 4°C, 30 min). The supernatant was collected and preserved at −80°C until the measurement of serum biochemical indices and hormones. The hepatopancreas and the ovaries of three crayfish in each container were randomly sampled and immediately frozen in liquid nitrogen and stored at −80°C for quantitative real-time PCR (qRT-PCR). The hepatopancreas (0.5 cm × 0.5 cm × 0.5 cm), midintestine (1.0 cm length), and unbroken ovary were separated and preserved in 4% paraformaldehyde for tissue section. The isolated abdominal muscle was weighed and stored at −40°C to calculate flesh content and determine proximate composition. These samples were stored at −40°C. Three crayfish were randomly sampled from each container and stored at −40°C to determine amino acid profile and proximate composition of the whole body. On the sampling day, 3hr after satiated feeding, the intestinal contents of three crayfish per container were separated and immediately frozen in liquid nitrogen and then stored at −80°C for the analysis of intestinal microbiota.

### 2.5. Analytic Methods

The standard methodology was used to determine the proximate composition of diets, muscles, and whole crayfish (AOAC, 2023). Diets were dried at 105°C to a consistent weight according to AOAC method 930.15 to measure their moisture content. The moisture content of the whole crayfish and abdominal muscle was determined using a vacuum freeze dryer (Christ Beta 2-4 LD plus LT, Marin Christ Corporation, Osterode, Germany) for 72 hr. The crude protein was measured with an auto Kjeldahl system (Kjelflex K-360; BUCHI Labortechnik AG, Flawil, Switzerland) according to AOAC method 984.13. Crude lipid content was determined by petroleum ether Soxhlet extraction (Sox606, Hanon Advanced Technology Group Co., Ltd.) according to AOAC method 2003.05. The ash content was determined by calcination in a muffle furnace (SX-4-10, Nanbei Instrument Limited) for 10 hr at 550°C according to AOAC method 942.05.

The amino acid concentrations of the whole crayfish (0.2 g) were determined using an automatic amino acid analyzer (HITACHI L-8900, Tokyo, Japan). Our previous study detailed the method [24].

Serum biochemical parameters were evaluated via an automated biochemical analyzer (BX-3010, Sysmex Corporation, Tokyo, Japan). The methods were also described in our previous study [24]. The reagents for the tests were purchased from Sysmex Company (Tokyo, Japan).

Approximately 0.6 g of fresh hepatopancreas and intestine samples was homogenized using a hand-held homogenizer (Biaoma FJ-150, Shanghai, China) on ice after exposure to nine times the normal volume of saline. The homogenates were centrifuged under 3,000x *g* for 10 min at 4°C, and the supernatants were collected for further analysis. The total protease activity was determined using the Folin–phenol reagent method [25]. The lipase (cat: A054-2-1) and *α*-amylase (cat: C016-1-1) activities were quantified by the methyl halogenating substrate and starch iodine chromogenic methods, respectively. The total protein content (cat: A045-3) of the supernatant was quantified by the bicinchoninic acid (BCA) method. All kits were purchased from the Nanjing Jiancheng Bioengineering Institute (Nanjing, China).

The hepatopancreas, intestine, and ovarian samples were embedded and dehydrated using a sequence of xylene and alcohol solutions in paraffin before being cut into 5 *μ*m sections and stained with hematoxylin and eosin. The stained sections were observed using the light microscope DM2500 (Leica, Germany). The length and width of intestinal villi and the incidence of hepatopancreas cells (B cells and R cells) were assessed using the Image ProPlus 6.0 image analysis system.

The total antioxidant capacity (T-AOC, cat: A015-2-1), malondialdehyde content (MDA, cat: A003-1), superoxide dismutase activity (SOD, cat: A001-3), and catalase activity (CAT, cat: A007-1-1) were determined with commercial kits based on the ABTS method, the thiobarbituric acid (TBA) method, the water-soluble tetrazolium-1 (WST-1) method, and the ammonium molybdate method, respectively. The activities of acid phosphatase (ACP, cat: A060-2) and polyphenol oxidase (PPO, cat: A136-1-1) and the lysozyme content (LZM, cat: A050-1-1) were determined by the phenylene disodium phosphate method, phenol reaction method, and turbidimetric method, respectively. All kits were purchased from the Nanjing Jiancheng Bioengineering Institute.

The fresh hepatopancreas samples (about 1 g per sample) were accurately weighed and homogenized using a hand-held homogenizer (Biaoma FJ-150, Shanghai, China) with 9 mL of PBS at pH 7.2–7.4. The homogenized specimens were centrifuged (3,000x *g*, 10 min, 4°C). The supernatants were collected and divided into three copies for further testing. The serum vitellogenesis inhibitory hormone (VIH, cat: MM-102302), hepatopancreas ecdysteroid hormone (EH, cat: MM-9152902), and hepatopancreas second messenger cyclic adenosine monophosphate (cAMP, cat: MM-9105202) were detected using the double antibody sandwich method with specific antibodies. The detection was performed using a shrimp special ELISA kit manufactured by Jiangsu Meimian Industrial Co. Ltd. (Jiangsu Yancheng, China). The minimum detectable concentrations of VIH, EH, and cAMP were typically less than 1.6 ng/L, 0.7 ng/L, and 1 nmol/L, respectively. The inter- and intra-assay coefficients of variation were less than 10%.

This study analyzed the intestinal microflora of the control group (dietary threonine level is 7.16 g/kg) and the group with the best growth performance (dietary threonine level is 16.44 g/kg). Four samples were determined in each group. Approximately 0.6 g of mixed intestinal contents and mucosa samples was weighed for DNA extraction using the Fast DNA SPIN extraction kits (MP Biomedicals, Santa Ana, CA, US). The quality levels were assessed using a spectrophotometer (ND-1000, NanoDrop, DE, US). In brief, the 338F (5′-ACTCCTACGGGAGGCAGCA -3′) and 806R (5′-GGACTACHVGGGTWTCTAAT -3′) primers were used to amplify the V3–V4 region of the 16S rRNA gene for the analysis of intestinal microbiota [26]. The services of amplification and sequencing were provided by Shanghai Personal Biotechnology Co., Ltd. (Shanghai, China). The sequence reads were trimmed and assigned to each sample based on their unique barcodes. Sequences underwent quality filtering with fastp (0.19.6) followed by merging using FLASH (version 1.2.11). Then, chimeric sequences were removed from the datasets using the Quantitative Insights Into Microbial Ecology (QIIME, v1.8.0) pipeline. The sequences were clustered into operational taxonomic units (OTUs) at a 97% identity threshold. The RDP classifier (version 2.13, http://rdp.cme.msu.edu/) was used to classify and annotate each sequence. Species classification information was obtained through comparison with the SILVA 16S rRNA database (version 138) at a threshold of 70%. Bioinformatic analysis was conducted using the Majorbio Cloud platform (https://cloud.majorbio.com). The alpha diversity was analyzed by Mothur (version 1.30.2, https://www.arb-silva.de/) software, and the Chao1, Shannon, ACE, and Simpson indexes were calculated. According to the abundance data of phylum and genus level communities, the Student *T*-test evaluated the significant difference in species abundance, and the species with significant differences between groups were obtained.

The mRNA expression levels of genes in hepatopancreatic and ovarian samples were determined using the qRT-PCR method. The qRT-PCR was conducted on a quantitative thermal cycler (Light Cycler 480II, Roche, Basel, CH) with SYBR® Premix Ex Taq™ (Takara Bio, Dalian, China). The 18s rRNA served as a housekeeping gene. The primers used in the present study are listed in Table 3 and were synthesized by Sangon Bio-Tech Co., Ltd. (Shanghai, China). Each sample was tested in triplicate. The mRNA expression of genes was calculated using the 2^−*ΔΔ*CT^ method. The mRNA expression of each target gene in the control group was set as the reference value of 1. The qRT-PCR protocol used in this study has been previously reported [27].

### 2.6. Calculations and Statistical Analyses

(1) $$\begin{array}{c} \text{SR }\left(\text{survival rate},\%\right)=\left(\text{final crayfish number}/\text{initial crayfish number}\right)\times 100, \end{array}$$

(2) $$\begin{array}{c} \text{WGR }\left(\text{weight gain rate},\%\right)=\left(\text{final mean weight–initial mean weight}\right)/\text{initial mean weight}\times 100, \end{array}$$

(3) $$\begin{array}{c} \text{SGR }\left(\text{specific growth rate},\%/\text{d}\right)=\left[\ln\left(\text{final mean weight}/\text{initial mean weight}\right)\right]/56\times 100, \end{array}$$

(4) $$\begin{array}{c} \text{FCR }\left(\text{feed conversion ratio}\right)=\text{dry feed intake}/\text{wet weight gain}, \end{array}$$

(5) $$\begin{array}{c} \text{FI }\left(\text{feed intake},\text{g}/\text{shrimp}\right)=\text{dry feed intake}/\text{final crayfish number}, \end{array}$$

(6) $$\begin{array}{c} \text{HSI }\left(\text{hepatosomatic index},\%\right)=\text{hepatopancreas weight}/\text{crayfish body weight}\times 100, \end{array}$$

(7) $$\begin{array}{c} \text{FC }\left(\text{flesh percentage},\%\right)=\text{abdomen muscle weight}/\text{crayfish body weight}\times 100, \end{array}$$

(8) $$\begin{array}{c} \text{PER }\left(\text{protein efficiency ratio},\%\right)=\text{weight gain}/\text{crude protein intake}\times 100, \end{array}$$

(9) $$\begin{array}{c} \text{PDR }\left(\text{protein deposition rate},\%\right)=\text{protein gain}/\text{total protein consumption}\times 100, \end{array}$$

(10) $$\begin{array}{c} \text{amino acid deposition rate}\left(\%\right)=\text{amino acid gain}/\text{total amino acid consumption}\times 100, \end{array}$$

(11) $$\begin{array}{c} \text{GI}\left(\text{gonad index},\%\right)=\text{gonad weight}/\text{final mean weight}\times 100. \end{array}$$

The data were analyzed with SPSS 26.0 software (SPSS Inc., Chicago, IL, US). Results were presented as the mean ± S.D. (standard deviation). Normal distribution was tested using Shapiro–Wilk, and homogeneity of variance was analyzed using Levene's equal variance tests. Then, data were subjected to a one-way analysis of variance followed by Tukey's multiple range tests. In addition, data were analyzed to assess if the pattern (or trend) was linear or quadratic using orthogonal polynomial contrasts. The adjusted coefficient of determination (*R*^2^) was calculated and judged. Statistical differences were considered at *P* < 0.05. The regression analysis graphs were drawn using OriginLab 2019 (OriginLab Inc., Massachusetts, US).

## 3. Results

### 3.1. Growth Performance, Feed Utilization, and Protein Utilization

**Figure 1:**
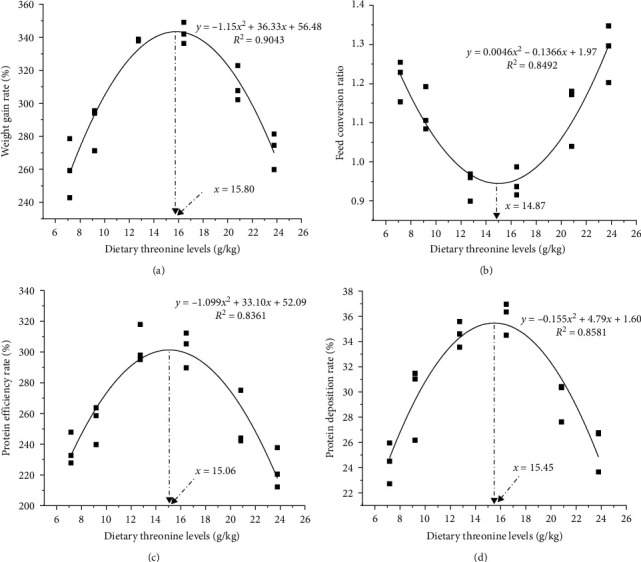
The quadratic curve model of the key indicators. The optimal dietary threonine levels based on weight gain rate (a), feed conversion ratio (b), protein efficiency rate (c), and protein deposition rate (d) of red swamp crayfish.

With increased dietary threonine levels, the WGR, SGR, and FCR showed a significant quadratic trend (*P* < 0.05). The WGR and SGR peaked at the 16.44 g/kg threonine group, and the FCR was lowest in the 12.74 g/kg threonine group. The PER and PDR showed a significant quadratic trend and reached their maximum in 12.74 and 16.44 g/kg threonine groups, respectively (Table 4). There was no significant difference in the FI, SR, HSI, and FC among the six groups (*P* > 0.05). Based on the quadratic curve model analysis of the relationship between WGR, FCR, PER, PDR, and dietary threonine levels, the optimal requirements of dietary threonine red swamp crayfish were 15.80, 14.87, 15.06, and 15.45 g/kg (dry matter), accounting for 45.16, 42.51, 43.05, and 44.16 g/kg of dietary protein, respectively (Figure 1).

### 3.2. Proximate Composition

Dietary threonine did not significantly affect the moisture content, crude lipid, and ash of the whole crayfish and abdominal muscle (*P* > 0.05) (Table 5). With the increased level of dietary threonine, the crude protein content of the whole crayfish and abdomen muscle showed a significant quadratic trend (*P* < 0.05), reaching their maximum at the levels of 12.74 and 16.44 g/kg threonine, respectively.

### 3.3. Amino Acid Analysis and Amino Acid Deposition Rates

With the increase in dietary threonine, the contents of arginine, lysine, methionine, phenylalanine, threonine, and valine in whole shrimp showed a significant quadratic trend (*P* < 0.05) (Table 6). Lysine and valine reached a maximum in the 12.74 g/kg group, whereas arginine, methionine, phenylalanine, and threonine reached a maximum in the 16.44 g/kg group. However, there were no significant effects on the contents of histidine, isoleucine, and leucine (*P* > 0.05). The contents of aspartic acid, proline, and tyrosine reached the maximum in the 12.74 g/kg group, and the contents of alanine and serine reached the maximum in the 16.44 g/kg group. There was no significant difference in the contents of glycine, glutamic acid, and cystine (*P* > 0.05). The *Σ*NEAA and *Σ*TAA contents peaked at 12.74 and 16.44 g/kg of dietary threonine, respectively (*P* < 0.05).

The deposition rates of arginine, histidine, isoleucine, lysine, methionine, phenylalanine, and valine showed a significant quadratic trend. In contrast, those of threonine and leucine showed a significant linear and quadratic trend (*P* < 0.05). In the 12.74 g/kg group, the deposition rates of threonine, histidine, lysine, and valine were significantly higher than those in the control group (Table 7). In the 16.44 g/kg group, the deposition rates of leucine, arginine, isoleucine, methionine, and phenylalanine were significantly higher than those in the control group (*P* < 0.05).

### 3.4. Serum Biochemical Indices

Dietary threonine had no significant effect on the contents of albumin (ALB), total cholesterol (T-CHO), and glucose (GLU) in the serum of crayfish (*P* > 0.05) (Table 8). Compared with the control group, the serum total protein (TP) content and alkaline phosphatase (ALP) activity showed a significant increase in the 12.74 g/kg group, and the triglyceride (TG) content and ACP activity showed a significant increase in the 16.44 g/kg group. The TP and TG contents and ALP activity in serum exhibited a significant quadratic trend, and ACP activity showed a significant linear and quadratic trend (*P* < 0.05). The aspartate aminotransferase (AST) and alanine transaminase (ALT) activities exhibited a significant quadratic trend (*P* < 0.05) and achieved their highest level in the 16.44 g/kg group.

### 3.5. Activities of Digestive Enzymes in Hepatopancreas and Intestine

Dietary threonine did not significantly affect *α*-amylase activity in the hepatopancreas and intestines (*P* > 0.05) (Table 9). Compared to the control group, the activities of lipase and protease in the hepatopancreas and intestines significantly increased when the threonine content was 12.74 and 16.44 g/kg, respectively. The activities of lipase and protease in the hepatopancreas and intestines showed a significant quadratic trend (*P* < 0.05).

### 3.6. Antioxidant Capacity and Nonspecific Immunity

With the increased levels of dietary threonine, the activities of T-AOC and SOD and the MDA content in the hepatopancreas showed a significant quadratic trend, and the CAT activities in the hepatopancreas showed a significant linear and quadratic trend (*P* < 0.05) (Table 10).

With the increased levels of dietary threonine, the T-AOC, SOD activities, and MDA content in hepatopancreas showed a significant quadratic trend and the CAT activities in hepatopancreas showed a significant linear and quadratic trend (*P* < 0.05) (Table 10). The T-AOC and SOD activity peaked at the 12.74 g/kg group, while the highest CAT activity and the lowest MDA content were observed in the 16.44 g/kg group. The T-AOC, SOD activity, and CAT activity in the intestine exhibited a significant linear and quadratic trend (*P* < 0.05). The CAT activity peaked at the 12.74 g/kg group, while the T-AOC and SOD activity peaked at the 16.44 g/kg group. The serum of LZM content showed a significant quadratic trend, and the serum of PPO activity showed a significant linear and quadratic change trend, reaching their highest levels in the 12.74 g/kg group and 16.44 g/kg group, respectively (*P* < 0.05).

### 3.7. Histological Observation in Hepatopancreas and Intestine

**Figure 2:**
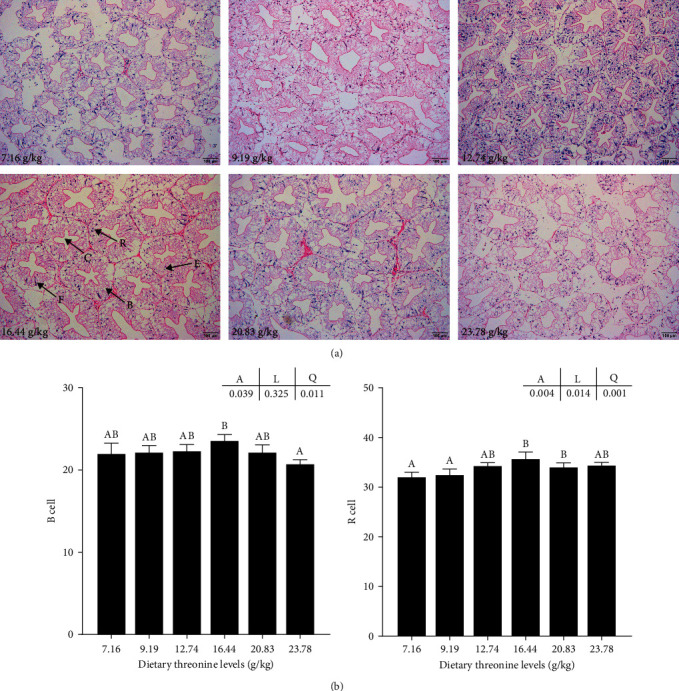
Effect of dietary threonine levels (dry matter basis, g/kg) on the histomorphology of hepatopancreas of red swamp crayfish. (a) B cells (B), E cells (E), F cells (F), R cells (R), and stellate lumen (C). The magnification is 100x. Scale bar = 100 *μ*m. (b) B-cell and R-cell prevalence (number/tubule) from the hepatopancreas of red swamp crayfish. Data were expressed as mean ± SD (*n* = 3). Different letters above the vertical bars represent significant differences (*P* < 0.05). A, *p*-value of the variance analyzed by one-way ANOVA; L, *p*-value of linear trend analyzed by orthogonal polynomial contrasts; and Q, *p*-value of quadratic trend analyzed by orthogonal polynomial contrasts.

The hepatopancreatic tubules of crayfish were closely arranged after dietary 12.74 and 16.44 g/kg of threonine (Figure 2(a)). The shape of cells was more regular, and the structure of the basement membrane was more complete. The lumen showed a regular star shape. However, the arrangement of hepatopancreatic tubules in the 7.16 and 23.78 g/kg groups was loose and atrophied to a certain extent, and the lumen was irregular. In addition, with the increase of dietary threonine, the proportion of B cells showed a significant quadratic trend and the proportion of R cells showed a significant linear and quadratic trend, and both of them reached a maximum in the 16.44 g/kg group (*P* < 0.05) (Figure 2(b)).

**Figure 3:**
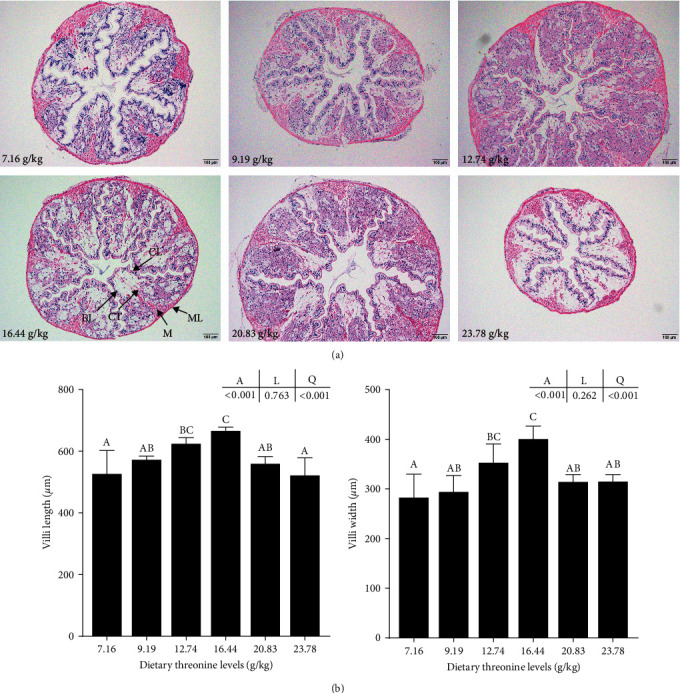
Effect of dietary threonine levels (dry matter basis, g/kg) on the histomorphology of intestinal of red swamp crayfish. (a) CL, chitin layer; EL, epithelium; CT, connective tissue layer; M, muscle bundle; and ML, muscle layer. The magnification is 100x. Scale bar = 100 *μ*m. (b) Effects of dietary threonine levels on the length and width of intestinal villi of red swamp crayfish. Data were expressed as mean ± SD (*n* = 3). Different letters above the vertical bars represent significant differences (*P* < 0.05). A, *p*-value of the variance analyzed by one-way ANOVA; L, *p*-value of linear trend analyzed by orthogonal polynomial contrasts; and Q, *p*-value of quadratic trend analyzed by orthogonal polynomial contrasts.

The intestinal histomorphology of crayfish from all groups was complete, with no deformities or pathological changes (Figure 3(a)). However, when the levels of threonine increased to 12.74 and 16.44 g/kg, the intestinal villi of crayfish were more neatly arranged. The small folds on the surface were more developed, the connective tissue density was improved, and the muscle bundles were complete. With the increase in dietary threonine levels, the length and width of intestinal villi exhibited a significant quadratic trend, which peaked at the 16.44 g/kg group (Figure 3(b)).

### 3.8. Microbial Diversity Analysis

**Figure 4:**
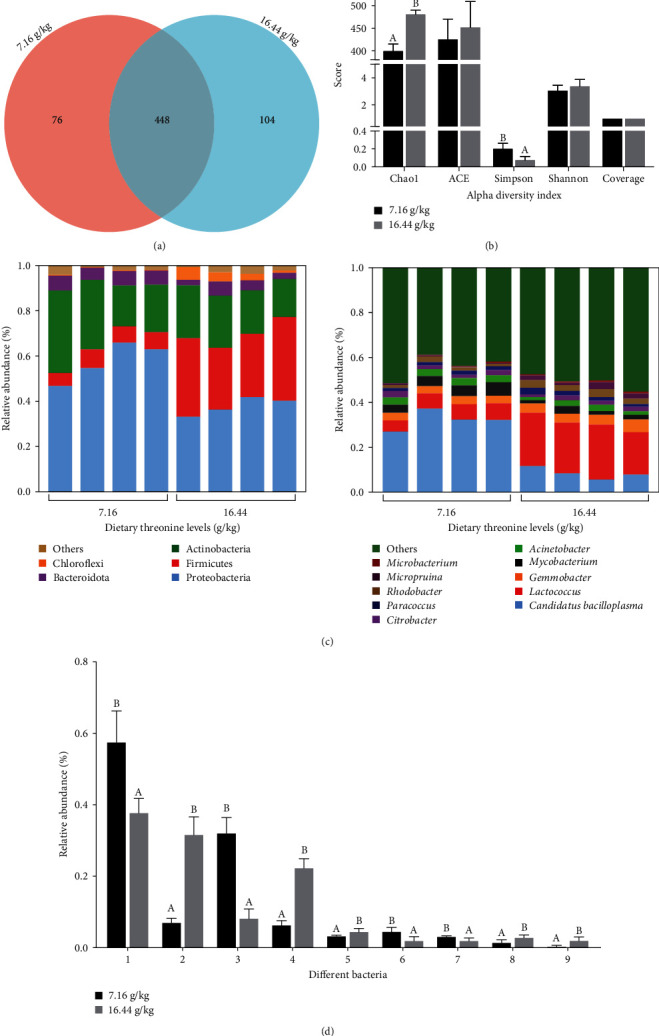
Effect of dietary threonine levels (dry matter basis, g/kg) on intestinal microflora of red swamp crayfish. (a) Venn diagram of the intestinal microbiota of red swamp crayfish (OTU level). (b) Alpha diversity of intestinal microbiota of red swamp crayfish. (c) Intestinal bacterial composition at the phylum and genus levels. Only the top 5 and 10 most abundant (based on relative abundance) bacterial phyla and genera were shown. Other phyla genera were all assigned as “others.” (d) Intestinal differential flora analysis of *P. clarkii*. 1. Proteobacteria; 2. Firmicutes; 3. *Candidatus bacilloplasma*; 4. *Lactococcus*; 5. *Gemmobacter*; 6. *Mycobacterium*; 7. *Acinetobacter*; 8. *Rhodobacter*; 9. *Micropruina*. Data were expressed as mean ± SD (*n* = 3). Different letters above the vertical bars represent significant differences (*P* < 0.05).

A total of 432,526 sequences were obtained from the analysis of intestinal 16S rDNA sequencing. Each sample was sequenced for more than 40,000 bp, with an average length of 418 bp. A total of 628 operational taxonomic units (OTUs) were identified (Figure 4(a)). The 7.16 g/kg group had 76 unique OTUs, and the 16.44 g/kg group had 104 unique OTUs. The library coverage of each sample was > 99.9%. The Chao1 index was higher, while the Simpson index was lower (*P* < 0.05) in the 16.44 g/kg group compared with the 7.16 g/kg group (Figure 4(b)). However, there were no significant differences in the ACE index or Shannon index (*P* > 0.05).

At the phylum level, the dominant intestinal microflora composition was similar between the two groups (Figures 4(c) and 4(d)). The relative abundance of Proteobacteria was the highest, followed by Firmicutes, Actinobacteria, Bacteroidota, and Chloroflexi. These five dominant florae accounted for 95% of the intestinal microflora. The 16.44 g/kg group had a higher abundance of Firmicutes and a lower abundance of Proteobacteria than those of the 7.16 g/kg group.

At the genus level, the predominant genera were *Candidatus Bacilloplasma*, *Lactococcus*, *Gemmobacter*, *Mycobacterium*, and *Acinetobacter* (Figures 4(c) and 4(d)). The 16.44 g/kg group had a higher abundance of *Lactococcus*, *Gemmobacter*, *Rhodobacter*, and *Micropruina* but lower abundance of *C. bacilloplasma*, *Mycobacterium*, and *Acinetobacter* compared to the 7.16 g/kg group (*P* < 0.05).

### 3.9. Regulatory Factors of Molting

**Figure 5:**
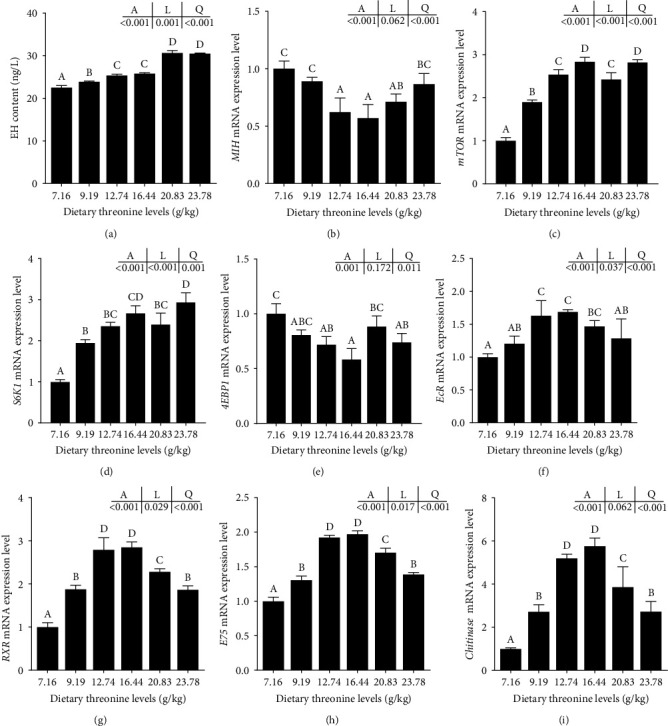
Effect of dietary threonine levels (dry matter basis, g/kg) on the regulatory factors of molting in the hepatopancreas of red swamp crayfish. (a) Ecdysterone hormone, EH; (b) molting inhibition hormone, *MIH*; (c) target of rapamycin, *mTOR*; (d) ribosomal protein S6 kinase 1, *S6K1*; (e) 4E binding protein 1, *4EBP1*; (f) ecdysone receptor, *EcR*; (g) retinoid X receptor, *RXR*; (h) *E75*; and (i) *chitinase*. Among them, (a) the content of EH in hepatopancreas and (b–i) the mRNA expression of *MIH*, *mTOR*, *S6K1*, *4EBP1*, *EcR*, *RXR*, *E75*, and *chitinase* genes in the hepatopancreas. Data were expressed as mean ± SD (*n* = 3). Different letters above the vertical bars represent significant differences (*P* < 0.05). A, *p*-value of the variance analyzed by one-way ANOVA; L, *p*-value of linear trend analyzed by orthogonal polynomial contrasts; and Q, *p*-value of quadratic trend analyzed by orthogonal polynomial contrasts.

Increasing dietary threonine resulted in a significant linear and quadratic trend (*P* < 0.05) in EH content in the hepatopancreas, which achieved its maximum at 20.83 g/kg group (Figure 5). Compared to the control group, the relative mRNA expressions of target of rapamycin (*mTOR*), ribosomal protein S6 kinase 1 (*S6K1*), 4E binding protein 1 (*4EBP*), ecdysteroid receptor (*EcR*), retinoid X receptor (*RXR*), *E75*, and *chitinase* in the hepatopancreas were significantly upregulating, and the expression of *4EBP1* and *MIH* was significantly downregulated in the 16.44 g/kg group (*P* < 0.05).

### 3.10. Regulatory Factors of Ovarian Development

**Figure 6:**
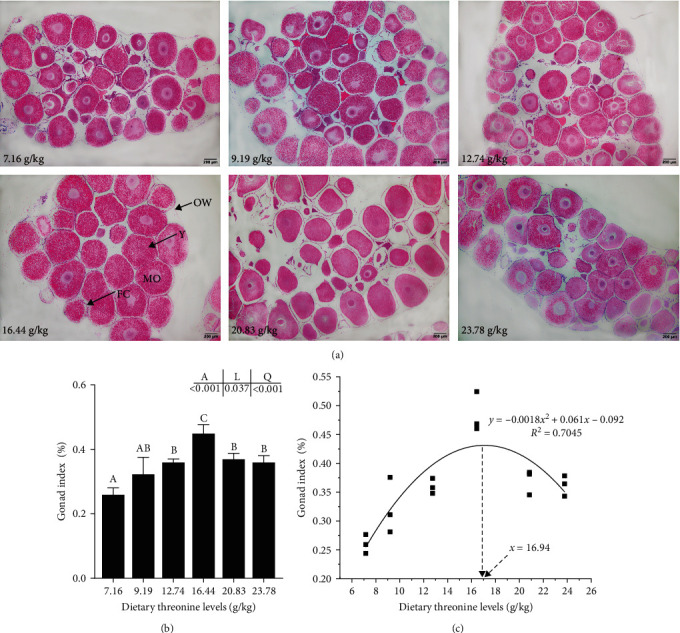
Effect of dietary threonine levels (dry matter basis, g/kg) on the ovarian development of red swamp crayfish. (a) Transverse sections of the ovarian from red swamp crayfish. Y, yolk granule; OW, ovary wall; MO, oocyte; and FC, follicular cell. The magnification is 40x. Scale bar = 200 *μ*m. (b) Effects of dietary threonine levels on the gonadal index of red swamp crayfish. (c) Quadratic curve model analysis of the relationship between dietary threonine levels and gonadal index of red swamp crayfish. Data were expressed as mean ± SD (*n* = 3). Different letters above the vertical bars represent significant differences (*P* < 0.05). A, *p*-value of the variance analyzed by one-way ANOVA; L, *p*-value of linear trend analyzed by orthogonal polynomial contrasts; and Q, *p*-value of quadratic trend analyzed by orthogonal polynomial contrasts.

The ovaries of crayfish in all groups had developed to the vitellogenesis stage, and the yolk granules were distributed to varying degrees in the oocytes. When the threonine level was 16.44 g/kg, most of the oocytes in the ovaries were full and the nucleus disappeared (Figure 6(a)). The gonadal index (GI) of female crayfish exhibited a linear and quadratic trend when the dietary threonine level increased and reached the maximum in the 16.44 g/kg group (*P* < 0.05) (Figure 6(b)). The quadratic curve regression model analysis based on the GI and dietary threonine levels revealed that the optimal requirement of dietary threonine for red swamp crayfish was 16.94 g/kg (dry matter), which accounted for 48.42 g/kg of diet protein (Figure 6(c)).

**Figure 7:**
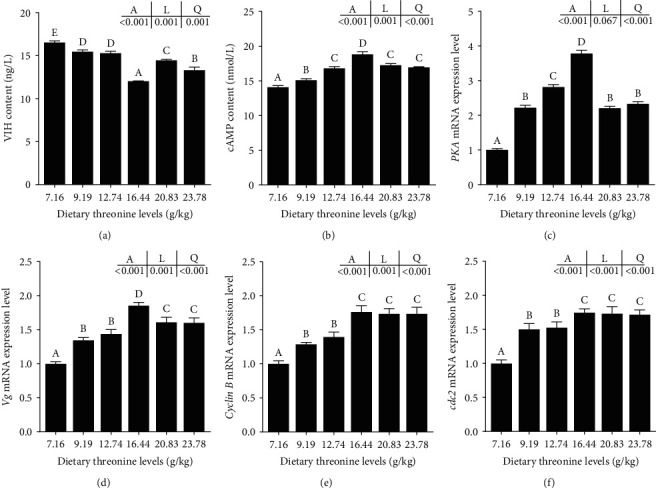
Effect of dietary threonine levels (dry matter basis, g/kg) on the regulatory factors of ovarian development of red swamp crayfish. (a) Vitellogenesis-inhibiting hormone, VIH; (b) cyclic adenosine monophosphate, cAMP; (c) protein kinase A, *PKA*; (d) vitellogenin, *Vg*; (e) *cyclin B*; and (f) cell division cycle 2, *cdc2*. Among them, (a–b) the VIH content in the serum and cAMP content in the hepatopancreas and (c–f) the mRNA expression of *PKA*, *Vg*, *cyclin B*, and *cdc2* genes in the hepatopancreas. Data were expressed as mean ± SD (*n* = 3). Different letters above the vertical bars represent significant differences (*P* < 0.05). A, *p*-value of the variance analyzed by one-way ANOVA; L, *p*-value of linear trend analyzed by orthogonal polynomial contrasts; and Q, *p*-value of quadratic trend analyzed by orthogonal polynomial contrasts.

Increasing dietary threonine resulted in a significant linear and quadratic trend in the content of VIH in serum and cAMP in hepatopancreas (*P* < 0.05) (Figure 7). Compared with the control group, the relative expression of protein kinase A (*PKA*) and vitellogenin (*Vg*) in hepatopancreas and *cyclin B* and cell division cycle 2 (*cdc2*) in the ovary was significantly upregulating in the 16.44 g/kg group (*P* < 0.05).

## 4. Discussion

### 4.1. Dietary Threonine Promoted the Molting and Growth Performance of Crayfish

The present study revealed that both deficiency and excess of dietary threonine negatively impacted the growth and feed utilization of red swamp crayfish but did not affect survival. These findings are consistent with previous studies on white shrimp [3, 15], Chinese mitten crab [16], and black tiger shrimp [17]. The optimal dietary threonine requirement for red swamp crayfish was 14.87–15.80 g/kg (dry matter) of diet, corresponding to 42.51–45.16 g/kg of dietary protein. This is similar to the threonine requirement of Chinese mitten crab (15.90 g/kg) [16] and black tiger shrimp (14.00 g/kg) [17] but higher than white shrimp (11.80 g/kg) [3]. The study also revealed differences in dietary threonine requirements among different animals.

Crustaceans shed their old shells periodically and form new ones for survival, growth, development, and reproduction [4]. The EH exerts its physiological function mainly through the ecdysone signal transduction pathway [28]. In the classical ecdysone signaling pathway, EH activates the heterodimer formed by the ecdysone receptor (*EcR*) and the retinoic acid X receptor (*RXR*) after entering the nucleus. The compound binds to EH and activates downstream transcription factors (*E75*) to promote molting [28]. *mTOR* is an atypical serine/threonine protein kinase [29] and is the main regulatory pathway for activating Y-organs (YO) to release EH [30]. Previous studies have reported that threonine could regulate the *mTOR* signal pathway [12, 13]. Our findings indicated that dietary threonine activated the ecdysone signal pathway (12.74–16.44 g/kg) and the *mTOR* pathway (16.44 g/kg) to promote molting by upregulating the mRNA expression levels of *EcR*, *RXR*, *E75*, *mTOR*, and *S6K1*. In addition, after synthesis in X-organs, *MIH* mainly acts on YO and inhibits EH synthesis [31]. In this experiment, threonine promoted EH synthesis by downregulating the mRNA expression of *MIH*. However, whether threonine directly affects *MIH* release remains to be confirmed [30]. After molting, a significant amount of chitin is required to synthesize a new exoskeleton [32]. In this experiment, it was hypothesized that threonine (16.44 g/kg) increased chitinase secretion, leading to the synthesis of new exoskeleton and shortened molting cycle for red swamp crayfish. These results indicated that threonine may activate the ecdysone through the *MIH* and *mTOR* pathways, regulate periodic molting, and promote growth performance.

### 4.2. Dietary Threonine Improved the Protein Utilization and Digestion Function of Crayfish

Threonine is a precursor for protein synthesis and a critical signal molecule that regulates the protein synthesis pathway [10]. Dietary threonine can activate the PI3K/AKT/TOR/*S6K1* signaling pathway to promote protein synthesis [12]. The present experiment showed that dietary threonine (12.74–16.44 g/kg) increased body protein content, protein efficiency, and protein deposition rate by activating the *mTOR* pathway. The results are consistent with studies on hybrid groupers (*Epinephelus fuscoguttatus*♀ × *Epinephelus lanceolatus*♂) [12], Indian carp (*Catla catla*) [33], and grass carp [34]. However, dietary threonine levels did not affect the body protein content in Chinese mitten crab [16] and white shrimp [3]. This may be related to the ability of different species to maintain body stable protein level.

Deficiencies of individual amino acids in the diet cause an imbalance in the ratio of EAAs, which affects the absorption and utilization of other amino acids [1]. Studies on white shrimp [15] and Atlantic salmon (*Salmo salar*) [35] have found that dietary threonine could significantly affect the level of amino acids in the muscle and whole body. This study showed that optimal dietary threonine (12.74–16.44 g/kg) significantly increased the contents of EAAs in whole crayfish. However, the deficiency (7.16 g/kg) or excess (23.78 g/kg) of dietary threonine decreased amino acid content. This may be due to nutritional antagonism caused by imbalanced dietary EAAs [9]. Studies on juvenile grass carp showed that the maximum threonine deposition rate of the whole body was observed in the threonine deficiency (7.30 g/kg) group [34]. Different from the above studies, both deficiency and excess of dietary threonine decreased the threonine deposition rate in our study, which may be due to different species. The findings demonstrated that an appropriate level of dietary threonine (12.74–16.44 g/kg) could balance dietary amino acids and improve the deposition of EAAs, ultimately leading to increased protein deposition.

The utilization of dietary nutrients is closely related to the digestion and absorption function. The hepatopancreatic tubule is the fundamental structural unit responsible for digestion and absorption in the crayfish hepatopancreas [36]. Threonine was beneficial in promoting the development of hepatopancreas in blunt snout bream [1]. A significant increase in incidence of B and R cells in hepatopancreatic tubules was observed after feeding 16.44 g/kg of dietary threonine. The intestinal tract is another important organ for crustaceans to perform digestion and absorption [37]. Our findings indicated that threonine (12.74–16.44 g/kg) promoted the development of intestinal villi, thereby increasing the surface area available for feed digestion and absorption within the intestine. The results align with previous studies on hybrid groupers [12] and grass carp [34]. This indicates that adequate threonine is taken and utilized by the intestinal tract and contributes to the maintenance of intestinal tract integrity. The activity of digestive enzymes is closely related to the strength of digestive function [38]. Our study revealed that dietary threonine increased the protease and lipase activities in the hepatopancreas and intestines of red swamp crayfish, which was consistent with the results in the Chinese mitten crab [16].

The composition and abundance of intestinal microflora are important factors that affect nutrition absorption, energy balance, and immune response of the host [39]. Threonine can regulate the imbalance of intestinal microflora and increase the abundance of beneficial microflora [10]. In this experiment, dietary threonine significantly improved the diversity of intestinal microflora. This may be due to the interaction between threonine and dietary components, enhancing intestinal microflora fermentation and promoting microflora colonization [10]. Threonine led to a significant increase in the relative abundance of Firmicutes, which was directly related to the increase in *Lactococcus* abundance. Additionally, the relative abundance of Proteobacteria decreased, which was mainly due to the decrease in *Acinetobacter* abundance. *Lactococcus* metabolites can promote the metabolism and absorption of nutrients [40] and degrade undigested amino acids into short-chain fatty acids [41]. This may explain the improved intestinal digestion of red swamp crayfish. These results indicate that threonine can promote protein synthesis by activating the *mTOR* signaling pathway. On the other hand, threonine can promote the development of hepatopancreas and intestinal tissues, increase the abundance of beneficial intestinal microflora, increase digestive and absorption functions, and ultimately improve feed utilization of crayfish.

### 4.3. Dietary Threonine Improved the Antioxidant Capacity and Nonspecific Immune Function of Crayfish

The antioxidant system is associated with the health status and immune system of aquatic animals [37]. Studies have demonstrated that dietary threonine deficiency can cause oxidative stress damage in tissues and cells [3, 13, 14, 42]. In this study, a threonine deficiency (7.16 g/kg) significantly increased the content of hepatopancreas MDA and the activities of serum AST and ALT, indicating that threonine deficiency could lead to hepatopancreatic injury. Optimal levels of dietary threonine can enhance antioxidant enzyme activity and reduce oxidative stress in animals [10]. Our finding indicated that the diet containing 12.74–16.44 g/kg of dietary threonine could significantly improve the antioxidant capacity of the crayfish by increasing the T-AOC and activities of SOD and CAT in the hepatopancreas and intestine, which was consistent with the results in white shrimp [3] and Chinese mitten crab [16]. These results suggest that threonine can enhance the antioxidant ability of red swamp crayfish by enhancing the activity of antioxidant enzymes, thus reducing oxidative stress damage.

Crustaceans have a relatively well-developed nonspecific immune system, in which the prophenoloxidase (proPO) system plays a crucial role [43]. Threonine can activate the proPO system of white shrimp and increase the activity of polyphenol oxidase (PPO) in serum to enhance immune function [3]. The findings of the present study were in accordance with these observations. In addition, threonine significantly increased the serum ACP, ALP, and LZM activities, thereby enhancing the immune response of red swamp crayfish. These results align with previous studies on Chinese mitten crabs [16]. Overall, the optimal levels of dietary threonine (12.74–16.44 g/kg) enhanced the nonspecific immune function of red swamp crayfish by regulating the antioxidant capacity and immune enzyme activity.

### 4.4. Dietary Threonine Promoted the Ovarian Development of Crayfish

The process of ovarian development in crustaceans involves oocyte development and yolk deposition [6]. Studies have shown that *cyclin B* and *cdc2* genes are highly expressed in the early stage of ovarian development (II stage) of red swamp crayfish during the meiotic maturation of oocytes [44]. During the early stages of vitellogenesis (III stage) and vitellogenesis (IV stage), the oocytes accumulate a large amount of nutrients, while the meiosis activity and the expression levels of *cyclin B* and *cdc2* were decreased [45]. During the ovary development and maturity stage (V stage), the yolk accumulates to a certain extent, promoting oocyte development and maturation by resuming meiosis [45]. In the present study, the ovaries of all groups had reached stage IV, and the relative expression of *cyclin B* and *cdc2* genes stabilized when the levels of dietary threonine exceeded 16.44 g/kg, suggesting that the appropriate amount of dietary threonine (16.44 g/kg) could promote the ovarian development of crayfish.

The deposition of yolk substance is the critical component of ovarian development and is a determining factor in the transition of the ovary from the vitellogenesis stage (IV stage) to the mature stage (V stage) [6]. The level of *Vg* directly affected the deposition of yolk substance, which is mainly synthesized in the hepatopancreas of red swamp crayfish [37]. The synthesis of *Vg* is regulated by the hyperglycemic hormone (CHH) family [46] and nutrients including amino acids [47]. The VIH is synthesized in the X-organ-sinus gland of the eyestalk and has been proven to inhibit *Vg* synthesis in giant river prawns (*Macrobrachium rosenbergii*) [46] and white shrimp [48]. Protein nutrition has been proven to promote *Vg* synthesis [48], but the underlying mechanism is unclear. The nutritional studies of Chinese mitten crabs suggest that arginine may regulate *Vg* synthesis through the VIH-cGMP pathway [49]. In this study, the VIH content in serum decreased with the increase in dietary threonine levels, reaching its minimum at the threonine level of 16.44 g/kg. Dietary threonine upregulating the *Vg* level by reducing the VIH level and increasing *Vg* expression, which was mediated by an increase in the second messenger cAMP and the downstream regulatory factor PKA [5, 50]. The difference may be due to differences in the second messenger of the hormone in different crustaceans or the way different amino acids regulate the hormone. In addition, it has been reported that threonine can regulate the expression of the *Vg* gene in silkworms (*B. mori*) through the *mTOR* signal pathway [18]. Our finding suggested that threonine may regulate vitellogenin synthesis through the *mTOR* signaling pathway. Based on the results of this experiment, it is hypothesized that threonine might regulate the expression of *Vg* in the hepatopancreas through the VIH-cAMP pathway and *mTOR* pathway, promoting vitellin synthesis and explaining the increase in GI of red swamp crayfish.

## 5. Conclusion

The optimal dietary threonine (12.74–16.44 g/kg) promoted the growth performance and nonspecific immune function of red swamp crayfish by improving feed protein utilization, digestive organ development, molting, antioxidant capacity, and immune enzyme activity. Dietary threonine regulated yolk substance deposition by decreasing serum VIH level and increasing *Vg* mRNA expression, thus promoting ovarian development in crayfish. Dietary threonine requirement of red swamp crayfish, determined by the WGR, FCR, PDR, and GI, was 14.87–16.94 g/kg (dry matter), accounting for 42.51–48.42 g/kg of dietary protein.

## Figures and Tables

**Table 1:** Composition of the experimental diets (dry matter basis, g/kg).

Ingredients	Threonine levels (dry matter basis, g/kg)					
	7.16	9.19	12.74	16.44	20.83	23.78
Fish meal	100.00	100.00	100.00	100.00	100.00	100.00
Wheat gluten	85.00	85.00	85.00	85.00	85.00	85.00
Peanut meal	250.00	250.00	250.00	250.00	250.00	250.00
Wheat flour	280.00	280.00	280.00	280.00	280.00	280.00
Sodium alginate	20.00	20.00	20.00	20.00	20.00	20.00
Ca (H_2_PO_4_)_2_	25.00	25.00	25.00	25.00	25.00	25.00
Yeast	20.00	20.00	20.00	20.00	20.00	20.00
Fish oil	25.00	25.00	25.00	25.00	25.00	25.00
Soybean oil	25.00	25.00	25.00	25.00	25.00	25.00
Soybean lecithin	10.00	10.00	10.00	10.00	10.00	10.00
Vitamin premix^1^	10.00	10.00	10.00	10.00	10.00	10.00
Cholesterol	5.00	5.00	5.00	5.00	5.00	5.00
Mineral premix^2^	10.00	10.00	10.00	10.00	10.00	10.00
Vitamin C	3.00	3.00	3.00	3.00	3.00	3.00
Choline chloride	2.00	2.00	2.00	2.00	2.00	2.00
Chitosan	1.00	1.00	1.00	1.00	1.00	1.00
Astaxanthin	0.40	0.40	0.40	0.40	0.40	0.40
Bentonite	40.00	40.00	40.00	40.00	40.00	40.00
Microfiber	66.30	66.30	66.30	66.30	66.30	66.30
L-Lys	6.00	6.00	6.00	6.00	6.00	6.00
L-His	1.80	1.80	1.80	1.80	1.80	1.80
DL-Met	2.00	2.00	2.00	2.00	2.00	2.00
L-Thr	0.00	2.50	5.00	7.50	10.00	12.50
L-Ala	12.50	10.00	7.50	5.00	2.50	0.00

^1^Per kg of vitamin premix contains vitamin A 4 g, vitamin D 0.02 g, vitamin E 10 g, vitamin K_3_ 10 g, vitamin B_1_ 10 g, vitamin B_2_ 10 g, vitamin B_6_ 20 g, nicotinic acid 40 g, biotin 0.2 g, calcium pantothenate 20 g, folic acid 0.5 g, vitamin B_12_ 0.01 g, vitamin C 20 g, and inositol 400 g; all ingredients were diluted with microcellulose to 1 kg. ^2^Per kg of mineral premix contains KIO_3_ 0.6 g, Na_2_SeO_3_·5H_2_O 0.08 g, KH_2_PO_4_ 320 g, MgSO_4_ 200 g, MnSO_4_·H_2_O 20 g, CuCl_2_·2H_2_O 2 g, ZnSO_4_·7H_2_O 60 g, FeSO_4_·7H_2_O 50 g, NaCl 100 g, and CoCl_2_ · 6H_2_O 2 g; all ingredients were diluted with microcellulose to 1 kg.

**Table 2:** Proximate composition and amino acid profile of the experimental diets (dry matter basis, g/kg).

Nutrient composition	Threonine levels (dry matter basis, g/kg)					
	7.16	9.19	12.74	16.44	20.83	23.78
Proximate composition						
Dry matter	934.07	939.83	937.84	936.24	933.71	936.72
Crude protein	349.83	346.48	343.17	347.07	344.36	349.59
Crude lipid	67.35	66.40	67.26	67.82	67.25	66.63
Ash	90.62	91.67	91.86	90.76	91.39	90.91
Gross energy^1^ (kJ/g)	18.24	18.33	18.40	18.23	18.37	18.35
Essential amino acids						
Arginine	13.43	13.15	13.20	13.36	13.48	13.87
Histidine	4.86	4.92	4.87	4.99	5.17	5.11
Isoleucine	6.63	6.54	6.61	6.47	6.55	6.85
Leucine	5.57	5.16	5.60	5.33	5.38	5.65
Lysine	12.92	12.76	12.63	13.10	12.9	13.08
Methionine	3.23	3.21	3.44	3.54	3.40	3.17
Phenylalanine	9.46	9.25	9.54	9.61	9.84	10.09
Threonine	7.16	9.19	12.74	16.44	20.83	23.78
Valine	10.16	10.15	9.77	10.68	10.51	10.65
Nonessential amino acids						
Alanine	18.73	15.69	13.12	12.66	10.24	8.31
Aspartic acid	29.24	29.42	30.05	30.03	30.20	29.54
Cysteine	2.06	2.02	2.07	2.46	1.99	2.05
Glycine	16.69	16.07	16.33	16.36	16.08	16.15
Glutamate	70.89	69.65	69.72	68.48	69.32	70.82
Proline	30.74	30.33	30.76	30.40	30.67	30.5
Serine	14.37	14.5	13.83	14.10	14.67	13.97
Tyrosine	12.89	12.83	12.81	12.98	13.04	13.01
Essential amino acids	73.42	74.33	78.4	83.52	88.06	92.25
Nonessential amino acids	195.61	190.51	188.69	187.47	186.21	184.35
Total amino acids	269.03	264.84	267.09	270.99	274.27	276.60

^1^Energy was determined by direct combustion in an adiabatic bomb calorimeter (SDC311, Hunan Sundy Science and Technology Development Co., Ltd., Changsha, Hunan Province, China).

**Table 3:** Nucleotide sequences of primers and cycling conditions used for PCR amplification.

Primer	Orientation	Primer sequence (5′ to 3′)	Length	Tm	Accession
Ovarian development regulators					
*Vg*	Forward	CCAGAAGACGCCACAAGAA	170	54.2	KR135171
	Reverse	CAGAAGGCATCAGCCAATC			
*PKA*	Forward	TACGCCATGAAGATACTCGACA	105	55.4	MW349851.1
	Reverse	GAGGAACGGGAAACTGATAGC			
*Cyclin B*	Forward	CCTTCTCTACTCAGTGCCTCG	130	57.0	KR135175
	Reverse	CGTACCCTGCTTCTTGCC			
*cdc2*	Forward	AATGGAGGACTACCTACGC	148	53.6	KR135176
	Reverse	GATAGCAGTGGATGGAACAC			
Molting regulatory factor					
*mTOR*	Forward	GAAGGCATGCTGCGGTATTG	122	57.5	XM045728234.1
	Reverse	CGCAGGCTTTGGGTCTCTTA			
*S6K1*	Forward	ACAGCCGAGAATCGCAAGAA	153	57.3	XM027373682.1
	Reverse	ATCACCATTATCGGGTCCGC			
*4EBP1*	Forward	ACCTGCCAGTGATACCAGGA	80	57.8	MW687141.1
	Reverse	TGGCTCCTCTGAAATCGTTCC			
*E75*	Forward	TGTCTACGACGCCATTAGGC	170	57.6	JQ350827.1
	Reverse	CGAATCTGCGATGTCCACCT			
*EcR*	Forward	GCTCGGACGCAGAGATTCAA	165	57.8	KX673814.1
	Reverse	GAAAGTTTTCGCCGCCGATG			
*MIH*	Forward	CTCCCAAGATCACAGCGTCA	214	57.2	MK820025.1
	Reverse	CAGTTCAAGGTCGAGTCCCA			
*RXR*	Forward	CACAAGTTTCCAGCCCAAGC	118	57.7	JX003647.1
	Reverse	GCTTGGCCCATTCTACCAGT			
*Chitinase*	Forward	TTTGACTCGGTGGGTGCT	385	55.8	JQ964138.1
	Reverse	TGTATGGTCCAGGCTTTCC			
Reference gene					
*18 s rRNA*	Forward	TCCGCATCACACTCACGT	165	55.9	KR135172
	Reverse	TGGAACCCTCTCCACAGG			

*Vg*, vitellogenin; *PKA*, protein kinase A; *cdc2*; cell division cycle 2; *mTOR*, target of rapamycin; *S6K1*, ribosomal protein S6 kinase 1; *4EBP1*, 4E binding protein 1; *EcR*, ecdysteroid receptor; *RXR*, retinoid X receptor; and *MIH*, molt-inhibiting hormone.

**Table 4:** Effect of dietary threonine level on growth performance of red swamp crayfish.

Parameters	Threonine levels (dry matter basis, g/kg)						ANOVA	L		SOP	
	7.16	9.19	12.74	16.44	20.83	23.78	*p*-Value	Adj. *R^2^*	*p*-Value	Adj. *R^2^*	*p*-Value
IBW (g)	5.40 ± 0.06	5.48 ± 0.06	5.46 ± 0.08	5.41 ± 0.11	5.43 ± 0.06	5.41 ± 0.10	0.837	−0.045	0.609	−0.091	0.751
FBW (g)	19.46 ± 0.97^a^	21.19 ± 0.93^ab^	23.95 ± 0.36^c^	23.96 ± 0.30^c^	22.31 ± 0.58^bc^	20.13 ± 0.91^a^	<0.001	−0.045	0.617	0.867	<0.001
SR (%)	87.04 ± 3.21	90.74 ± 3.20	87.04 ± 3.21	87.03 ± 3.46	87.03 ± 3.54	87.03 ± 3.43	0.657	−0.018	0.416	−0.085	0.722
WGR (%)	260.17 ± 17.91^a^	286.91 ± 13.59^ab^	338.40 ± 6.56^c^	342.37 ± 6.37^c^	310.87 ± 10.72^bc^	271.86 ± 11.02^a^	<0.001	−0.038	0.549	0.892	<0.001
SGR (%/d)	2.29 ± 0.09^a^	2.42 ± 0.06^ab^	2.64 ± 0.05^c^	2.65 ± 0.03^c^	2.52 ± 0.04^bc^	2.35 ± 0.05^a^	<0.001	−0.035	0.521	0.885	<0.001
FCR	1.21 ± 0.05^bc^	1.12 ± 0.06^b^	0.94 ± 0.04^a^	0.95 ± 0.04^a^	1.13 ± 0.08^b^	1.28 ± 0.08^c^	<0.001	−0.024	0.451	0.827	<0.001
FI (g/shrimp)	19.78 ± 0.96	18.87 ± 0.32	19.98 ± 0.74	19.88 ± 0.89	20.33 ± 0.87	19.57 ± 0.83	0.390	−0.010	0.373	−0.039	0.522
HSI (%)	7.64 ± 0.27	7.17 ± 0.29	7.48 ± 0.19	7.43 ± 0.14	7.27 ± 0.17	7.43 ± 0.02	0.151	−0.040	0.568	−0.084	0.716
FC (%)	11.37 ± 0.09	10.77 ± 0.87	10.34 ± 0.37	10.82 ± 0.81	11.31 ± 0.63	11.61 ± 0.54	0.190	0.030	0.234	0.266	0.038
PER (%)	236.12 ± 10.45^a^	254.02 ± 12.58^a^	303.66 ± 12.41^b^	302.41 ± 11.54^b^	253.75 ± 18.43^a^	223.49 ± 13.05^a^	<0.001	−0.039	0.554	0.815	<0.001
PDR (%)	24.39 ± 1.62^a^	29.55 ± 2.94^b^	34.58 ± 1.02^c^	35.92 ± 1.28^c^	29.46 ± 1.59^b^	25.71 ± 1.76^ab^	<0.001	−0.062	0.928	0.838	<0.001

Values with different superscripts in the same row are significantly different (*P* < 0.05). Data were expressed as mean ± SD (*n* = 3). L, linear trend; Adj. *R*^2^, adjusted R square; and SOP, second-order polynomial trend.

**Table 5:** Effect of dietary threonine level on whole shrimp and abdominal muscle composition of red swamp crayfish (g/kg, wet mass).

Parameters	Threonine levels (dry matter basis, g/kg)						ANOVA	L		SOP	
	7.16	9.19	12.74	16.44	20.83	23.78	*p*-Value	Adj. *R^2^*	*p*-Value	Adj. *R^2^*	*p*-Value
Whole body											
Moisture	682.44 ± 11.98	680.18 ± 26.28	685.55 ± 13.52	681.53 ± 7.35	683.62 ± 45.30	682.14 ± 29.21	0.992	−0.062	0.962	−0.132	0.994
Crude protein	118.52 ± 6.13^a^	120.61 ± 3.52^a^	125.00 ± 4.33^ab^	129.26 ± 3.38^b^	120.20 ± 5.03^a^	117.98 ± 4.94^a^	<0.001	−0.028	0.847	0.351	<0.001
Crude lipid	38.91 ± 0.79	39.44 ± 2.82	38.68 ± 2.53	38.21 ± 5.12	39.00 ± 4.66	39.66 ± 2.87	0.999	−0.061	0.891	−0.114	0.878
Ash	65.79 ± 1.11	64.15 ± 2.51	63.76 ± 7.04	64.48 ± 3.62	64.76 ± 3.65	66.29 ± 8.91	0.989	−0.057	0.785	−0.093	0.764
Abdominal muscle											
Moisture	772.36 ± 3.93	770.22 ± 3.51	770.03 ± 5.65	777.45 ± 5.20	773.71 ± 3.23	770.17 ± 2.75	0.273	−0.054	0.727	−0.029	0.484
Crude protein	176.62 ± 2.29^a^	176.93 ± 4.93^a^	185.19 ± 4.62^b^	184.18 ± 3.05^b^	176.84 ± 4.09^a^	176.30 ± 1.14^a^	<0.001	−0.025	0.693	0.382	<0.001
Crude lipid	4.25 ± 0.12	4.38 ± 0.17	4.25 ± 0.12	4.22 ± 0.11	4.31 ± 0.12	4.31 ± 0.14	0.518	−0.062	0.965	−0.099	0.794
Ash	13.13 ± 0.29	13.07 ± 0.31	13.37 ± 0.62	13.10 ± 0.18	13.17 ± 0.09	13.19 ± 0.22	0.911	−0.060	0.848	−0.121	0.920

Values with different superscripts in the same row are significantly different (*P* < 0.05). Data were expressed as mean ± SD (*n* = 3). L, linear trend; Adj. *R*^2^ adjusted R square; and SOP, second-order polynomial trend.

**Table 6:** Effect of dietary threonine level on amino acid composition of red swamp crayfish (g/kg, wet mass).

Amino acids	Threonine levels (dry matter basis, g/kg)						ANOVA	L		SOP	
	7.16	9.19	12.74	16.44	20.83	23.78	*p*-Value	Adj. *R^2^*	*p*-Value	Adj. *R^2^*	*p*-Value
Arginine	5.48 ± 0.20^a^	5.82 ± 0.17^ab^	6.49±0.31 b^c^	6.66 ± 0.39^c^	5.79 ± 0.31^ab^	5.69 ± 0.32^ab^	0.002	−0.059	0.824	0.601	<0.001
Histidine	1.73 ± 0.10	1.61 ± 0.09	1.86 ± 0.14	1.71 ± 0.11	1.72 ± 0.13	1.58 ± 0.06	0.095	−0.020	0.424	0.098	0.181
Isoleucine	3.74 ± 0.19	3.56 ± 0.08	3.80 ± 0.20	3.77 ± 0.15	3.86 ± 0.16	3.50 ± 0.14	0.103	−0.057	0.779	0.067	0.233
Leucine	3.07 ± 0.09	2.87 ± 0.05	3.07 ± 0.27	3.10 ± 0.14	3.19 ± 0.14	2.87 ± 0.11	0.111	−0.061	0.888	0.006	0.373
Lysine	7.78 ± 0.33^ab^	7.31 ± 0.11^ab^	8.07 ± 0.22^b^	7.99 ± 0.44^b^	7.73 ± 0.11^ab^	6.91 ± 0.67^a^	0.018	0.039	0.213	0.350	0.015
Methionine	1.86 ± 0.06^a^	1.87 ± 0.13^a^	2.11 ± 0.12^ab^	2.29 ± 0.05^b^	2.12 ± 0.13^ab^	1.82 ± 0.14^a^	0.001	−0.038	0.551	0.632	<0.001
Phenylalanine	3.58 ± 0.07^ab^	3.55 ± 0.14^ab^	3.98 ± 0.07^c^	4.03 ± 0.08^c^	3.80 ± 0.16^bc^	3.34 ± 0.20^a^	<0.001	−0.054	0.719	0.700	<0.001
Threonine	3.89 ± 0.43^a^	4.12 ± 0.19^ab^	5.14 ± 0.09^cd^	5.46 ± 0.08^d^	4.65 ± 0.14^bc^	4.46 ± 0.11^ab^	<0.001	0.075	0.143	0.774	<0.001
Valine	4.55 ± 0.11^a^	4.58 ± 0.30^ab^	5.29 ± 0.18^c^	5.28 ± 0.23^bc^	4.68 ± 0.45^abc^	4.53 ± 0.06^a^	0.005	−0.062	0.939	0.517	0.002
Alanine	3.89 ± 0.06^a^	4.19 ± 0.06^ab^	4.64 ± 0.14^bc^	4.76 ± 0.13^c^	3.96 ± 0.09^a^	4.25 ± 0.44^abc^	0.001	−0.051	0.684	0.380	0.011
Aspartic acid	10.23 ± 0.49^ab^	9.66 ± 0.43^a^	11.02 ± 0.43^b^	10.54 ± 0.22^ab^	10.44 ± 0.47^ab^	9.76 ± 0.54^a^	0.023	−0.060	0.859	0.223	0.059
Cysteine	1.04 ± 0.05	0.98 ± 0.03	1.09 ± 0.04	1.10 ± 0.07	1.12 ± 0.03	0.99 ± 0.07	0.160	−0.038	0.547	0.260	0.041
Glycine	4.87 ± 0.12	4.63 ± 0.10	5.00 ± 0.15	4.98 ± 0.13	4.92 ± 0.14	4.53 ± 0.31	0.260	−0.028	0.472	0.255	0.043
Glutamate	13.11 ± 0.52	12.96 ± 0.67	14.42 ± 0.63	13.67 ± 0.22	14.30 ± 0.70	13.13 ± 0.84	0.050	−0.017	0.412	0.219	0.062
Proline	4.42 ± 0.16^ab^	4.20 ± 0.04^a^	4.95 ± 0.14^b^	4.66 ± 0.18^ab^	4.70 ± 0.30^ab^	4.64 ± 0.50^ab^	0.063	0.063	0.162	0.152	0.113
Serine	4.69 ± 0.18^ab^	4.37 ± 0.04^ab^	4.63 ± 0.05^ab^	4.88 ± 0.04^b^	4.30 ± 0.28^a^	4.25 ± 0.34^a^	0.012	0.094	0.116	0.224	0.058
Tyrosine	5.59 ± 0.16^ab^	5.60 ± 0.11^ab^	5.93 ± 0.04^b^	5.79 ± 0.12^ab^	5.85 ± 0.27^ab^	5.29 ± 0.41^a^	0.039	−0.031	0.495	0.366	0.013
*Σ*EAA	35.77 ± 1.16^a^	35.36 ± 0.45^a^	39.73 ± 1.32^b^	39.84 ± 1.48^b^	37.62 ± 1.32^ab^	34.95 ± 1.42^a^	0.001	−0.061	0.898	0.636	<0.001
*Σ*NEAA	48.40 ± 2.44	46.27 ± 2.27	51.87 ± 1.73	49.11 ± 2.43	49.65 ± 2.44	47.45 ± 1.21	0.095	−0.060	0.845	0.080	0.210
*Σ*TAA	84.17 ± 3.45^ab^	81.62 ± 2.68^a^	91.60 ± 2.07^b^	88.95 ± 3.57^ab^	87.26 ± 3.67^ab^	82.4 ± 2.55^a^	0.011	−0.060	0.860	0.374	0.012

Values with different superscripts in the same row are significantly different (*P* < 0.05). Data were expressed as mean ± SD (*n* = 3). L, linear trend; Adj. *R*^2^, adjusted R square; and SOP, second-order polynomial trend. *Σ*EAA, total essential amino acids; *Σ*NEAA, total nonessential amino acids; *Σ*TAA, total amino acids.

**Table 7:** Effect of dietary threonine level on the essential amino acid deposition rate of red swamp crayfish (wet weight, %).

Parameters	Threonine levels (dry matter basis, g/kg)						ANOVA	L		SOP	
	7.16	9.19	12.74	16.44	20.83	23.78	*p*-Value	Adj. *R^2^*	*p*-Value	Adj. *R^2^*	*p*-Value
ArgDR	22.31 ± 1.24^a^	31.19 ± 2.15^b^	40.79 ± 2.50^c^	42.30 ± 5.18^c^	29.81 ± 2.61^ab^	24.71 ± 1.51^ab^	<0.001	−0.062	0.982	0.832	<0.001
HisDR	21.54 ± 2.06^ab^	23.12 ± 2.34^ab^	32.36 ± 4.57^c^	28.14 ± 1.77^bc^	24.23 ± 2.43^ab^	18.92 ± 1.09^a^	0.001	−0.027	0.466	0.656	<0.001
IleDR	41.40 ± 2.36^ab^	46.68 ± 2.13^bc^	54.87 ± 3.33^d^	55.73 ± 2.34^d^	50.78 ± 2.14^cd^	38.79 ± 2.17^a^	<0.001	−0.061	0.871	0.882	<0.001
LeuDR	38.89 ± 1.21^a^	45.65 ± 1.31^ab^	50.50 ± 5.21^bc^	54.28 ± 1.86^c^	49.68 ± 2.72^bc^	55.13 ± 2.60^c^	<0.001	0.538	<0.001	0.670	<0.001
LysDR	39.88 ± 2.10^b^	44.37 ± 1.17^b^	56.87 ± 3.16^c^	52.84 ± 2.34^c^	43.51 ± 2.41^b^	32.47 ± 3.98^a^	<0.001	0.036	0.219	0.877	<0.001
MetDR	19.50 ± 1.10^a^	25.43 ± 4.35^ab^	36.91 ± 2.70^cd^	42.56 ± 4.42^d^	32.08 ± 5.21^bc^	18.73 ± 3.36^a^	<0.001	−0.057	0.774	0.850	<0.001
PheDR	24.29 ± 0.77^a^	29.54 ± 1.50^b^	37.25 ± 2.11^c^	37.91 ± 2.58^c^	30.28 ± 1.75^b^	21.78 ± 1.39^a^	<0.001	−0.045	0.611	0.931	<0.001
ThrDR	31.36 ± 5.60^b^	32.75 ± 2.83^b^	36.02 ± 1.87^b^	30.42 ± 1.44^b^	17.07 ± 0.47^a^	12.53 ± 0.61^a^	<0.001	0.679	<0.001	0.881	<0.001
ValDR	28.29 ± 1.26^a^	35.97 ± 3.52^a^	48.52 ± 2.22^b^	44.59 ± 3.52^b^	33.94 ± 4.54^a^	28.51 ± 0.35^a^	<0.001	−0.048	0.643	0.791	<0.001

Values with different superscripts in the same row are significantly different (*P* < 0.05). Data were expressed as mean ± SD (*n* = 3). L, linear trend; Adj. *R*^2^, adjusted R square; and SOP, second-order polynomial trend. ArgDR, arginine deposition rate; HisDR, histidine deposition rate; IleDR, isoleucine deposition rate; LeuDR, leucine deposition rate; LysDR, lysine deposition rate; MetDR, methionine deposition rate; PheDR, phenylalanine deposition rate; ThrDR, threonine deposition rate; TrpDR, tryptophan deposition rate; ValDR, valine deposition rate.

**Table 8:** Effect of dietary threonine level on serum biochemical indices of red swamp crayfish.

Parameters	Threonine levels (dry matter basis, g/kg)						ANOVA	L		SOP	
	7.16	9.19	12.74	16.44	20.83	23.78	*p*-Value	Adj. *R^2^*	*p*-Value	Adj. *R^2^*	*p*-Value
TP (g/L)	67.77 ± 3.98^a^	72.78 ± 3.00^ab^	83.21 ± 0.81^c^	78.98 ± 3.05^bc^	76.81 ± 1.00^bc^	72.49 ± 2.00^ab^	<0.001	−0.005	0.352	0.691	<0.001
ALB (g/L)	1.39 ± 0.30	1.40 ± 0.28	1.46 ± 0.10	1.51 ± 0.04	1.51 ± 0.25	1.41 ± 0.19	0.963	−0.047	0.630	−0.068	0.640
AST (U/L)	22.33 ± 1.53^c^	15.00 ± 1.00^b^	12.33 ± 1.53^ab^	10.33 ± 0.58^a^	15.67 ± 0.58^b^	20.67 ± 3.06^c^	<0.001	−0.062	0.912	0.849	<0.001
ALT (U/L)	56.33 ± 3.06^c^	48.33 ± 2.08^b^	45.67 ± 1.52^ab^	41.33 ± 1.53^a^	50.00 ± 2.00^b^	56.33 ± 2.08^c^	<0.001	−0.058	0.797	0.833	<0.001
ALP (U/L)	8.00 ± 1.00^a^	9.33 ± 2.082^ab^	15.33 ± 0.58^c^	13.33 ± 1.53^bc^	9.67 ± 2.52^ab^	8.33 ± 2.08^a^	0.001	−0.061	0.876	0.576	0.001
ACP (U/L)	12.09 ± 0.42^a^	14.73 ± 0.95^b^	18.45 ± 0.99^c^	22.19 ± 1.03^d^	18.4 ± 0.85^c^	18.17 ± 0.36^c^	<0.001	0.383	<0.001	0.860	<0.001
TG (mmol/L)	0.65 ± 0.01^b^	0.54 ± 0.01^a^	0.54 ± 0.02^a^	0.58 ± 0.05^ab^	0.53 ± 0.02^a^	0.63 ± 0.03^b^	<0.001	−0.061	0.877	0.293	0.029
T-CHO (mmol/L)	0.14 ± 0.01	0.12 ± 0.02	0.13 ± 0.01	0.12 ± 0.03	0.12 ± 0.01	0.14 ± 0.01	0.222	−0.062	0.987	0.032	0.306
GLU (mmol/L)	2.23 ± 0.28	2.40 ± 0.22	2.41 ± 0.12	2.53 ± 0.11	2.48 ± 0.56	2.44 ± 0.27	0.871	0.003	0.320	−0.006	0.411

Values with different superscripts in the same row are significantly different (*P* < 0.05). Data were expressed as mean ± SD (*n* = 3). L, linear trend; Adj. *R*^2^, adjusted R square; and SOP, second-order polynomial trend. TP, total protein; ALB, albumin; AST, aspartate aminotransferase; ALT, alanine aminotransferase; ALP, alkaline phosphatase; ACP, acid phosphatase; TG, triglyceride; T-CHO, total cholesterol; GLU, glucose.

**Table 9:** Effect of dietary threonine levels on the activities of digestive enzymes in hepatopancreas and intestines of red swamp crayfish.

Parameters	Threonine levels (dry matter basis, g/kg)						ANOVA	L		SOP	
	7.16	9.19	12.74	16.44	20.83	23.78	*p*-Value	Adj. *R^2^*	*p*-Value	Adj. *R^2^*	*p*-Value
Hepatopancreas											
Protease (U/g prot)	62.82 ± 3.77^a^	71.93 ± 5.31^ab^	74.08 ± 1.23^b^	94.99 ± 2.50^c^	66.17 ± 3.66^ab^	65.15 ± 2.69^ab^	<0.001	−0.062	0.951	0.516	0.002
*α*-Amylase (U/mg prot)	1.06 ± 0.07	1.07 ± 0.05	1.05 ± 0.03	1.08 ± 0.06	1.08 ± 0.05	1.07 ± 0.01	0.968	−0.034	0.513	−0.101	0.804
Lipase (U/g prot)	8.02 ± 0.75^a^	10.42 ± 2.36^ab^	17.48 ± 0.81^c^	12.11 ± 1.03^b^	11.76 ± 0.49^b^	10.59 ± 0.38^ab^	<0.001	−0.044	0.600	0.442	0.005
Intestine											
Protease (U/g prot)	20.13 ± 0.89^a^	26.9 ± 0.67^b^	33.97 ± 1.37^c^	46.98 ± 3.87^d^	24.54 ± 0.66^ab^	20.09 ± 0.98^a^	<0.001	−0.062	0.924	0.736	<0.001
*α*-Amylase (U/mg prot)	2.04 ± 0.12	2.04 ± 0.08	2.03 ± 0.09	2.06 ± 0.10	2.03 ± 0.06	2.09 ± 0.09	0.964	−0.032	0.504	−0.091	0.750
Lipase (U/g prot)	11.05 ± 0.91^a^	12.02 ± 0.43^ab^	17.49 ± 0.57^c^	13.30 ± 0.12^b^	12.15 ± 0.87^ab^	12.10 ± 0.32^ab^	<0.001	−0.062	0.935	0.367	0.013

Values with different superscripts in the same row are significantly different (*P* < 0.05). Data were expressed as mean ± SD (*n* = 3). L, linear trend; Adj. *R*^2^, adjusted R square; and SOP, second-order polynomial trend.

**Table 10:** Effect of dietary threonine level on antioxidant capacity and nonspecific immunity of red swamp crayfish.

Parameters	Threonine levels (dry matter basis, g/kg)						ANOVA	L		SOP	
	7.16	9.19	12.74	16.44	20.83	23.78	*p*-Value	Adj. *R^2^*	*p*-Value	Adj. *R^2^*	*p*-Value
Hepatopancreas											
T-AOC (U/mg prot)	0.42 ± 0.07^a^	0.42 ± 0.05^a^	0.73 ± 0.02^b^	0.50 ± 0.09^a^	0.47 ± 0.09^a^	0.42 ± 0.02^a^	<0.001	−0.025	0.700	0.327	0.001
SOD (U/mg prot)	56.35 ± 0.87^a^	59.21 ± 3.04^ab^	70.80 ± 1.69^c^	61.60 ± 2.09^b^	58.00 ± 2.55^ab^	56.58 ± 2.40^a^	<0.001	−0.003	0.353	0.462	<0.001
CAT (U/mg prot)	2.43 ± 0.09^a^	2.86 ± 0.09^b^	3.08 ± 0.09^c^	4.37 ± 0.09^d^	3.18 ± 0.07^c^	3.22 ± 0.21^c^	<0.001	0.195	0.004	0.624	<0.001
MDA (nmol/mg prot)	11.60 ± 0.74^c^	9.47 ± 0.48^b^	6.42 ± 0.55^a^	6.10 ± 0.78^a^	8.80 ± 1.27^b^	10.08 ± 0.94^b^	<0.001	0.001	0.314	0.807	<0.001
Intestine											
T-AOC (U/mg prot)	0.87 ± 0.10^a^	1.16 ± 0.06^bc^	1.20 ± 0.09^bc^	1.46 ± 0.02^d^	1.27 ± 0.06^c^	1.15 ± 0.05^b^	<0.001	0.200	0.004	0.759	<0.001
SOD (U/mg prot)	48.61 ± 1.62^a^	50.38 ± 1.06^a^	55.51 ± 1.55^b^	62.55 ± 1.74^c^	56.57 ± 2.70^b^	55.16 ± 2.60^b^	<0.001	0.293	<0.001	0.721	<0.001
CAT (U/mg prot)	0.90 ± 0.08^a^	1.32 ± 0.05^b^	2.56 ± 0.03^d^	2.44 ± 0.08^d^	1.64 ± 0.10^c^	1.67 ± 0.10^c^	<0.001	0.083	0.049	0.782	<0.001
Serum											
LZM (*µ*g/mL)	2.24 ± 0.23^a^	2.32 ± 0.20^a^	2.86 ± 0.08^b^	2.60 ± 0.47^ab^	2.62 ± 0.21^ab^	2.42 ± 0.06^a^	0.002	0.019	0.202	0.288	0.001
PPO (U/mL)	52.22 ± 5.09^a^	63.11 ± 6.19^ab^	79.33 ± 7.69^c^	97.33 ± 4.81^d^	79.78 ± 6.84^c^	74.89 ± 3.36^bc^	<0.001	0.265	0.017	0.800	<0.001

Values with different superscripts in the same row are significantly different (*P* < 0.05). Data were expressed as mean ± SD (*n* = 3). L, linear trend; Adj. *R*^2^, adjusted R square; and SOP, second-order polynomial trend. T-AOC, total antioxidant capacity; SOD, superoxide dismutase; CAT, catalase; MDA, malondialdehyde; LZM, lysozyme; PPO, polyphenol oxidase.

## Data Availability

All data supporting this research article are available from the corresponding author on request.
